# Supplementary material for: Interpretable representation learning for 3D multi-piece intracellular structures using point clouds
Source: bioRxiv. 2024 Jul 27:2024.07.25.605164. Preprint. [Version 2] doi: 10.1101/2024.07.25.605164 (PMC11291148; doi:10.1101/2024.07.25.605164)
Supplement: Supplementary file 1 [file NIHPP2024.07.25.605164v2-supplement-1.pdf]

bioRxiv preprint doi: https://doi.org/10.1101/2024.07.25.605164; this version posted July 27, 2024. The copyright holder for this preprint (which was not certified by peer review) is the author/funder, who has granted bioRxiv a license to display the preprint in perpetuity. It is made available under aCC-BY 4.0 International license.

Supplemental figures

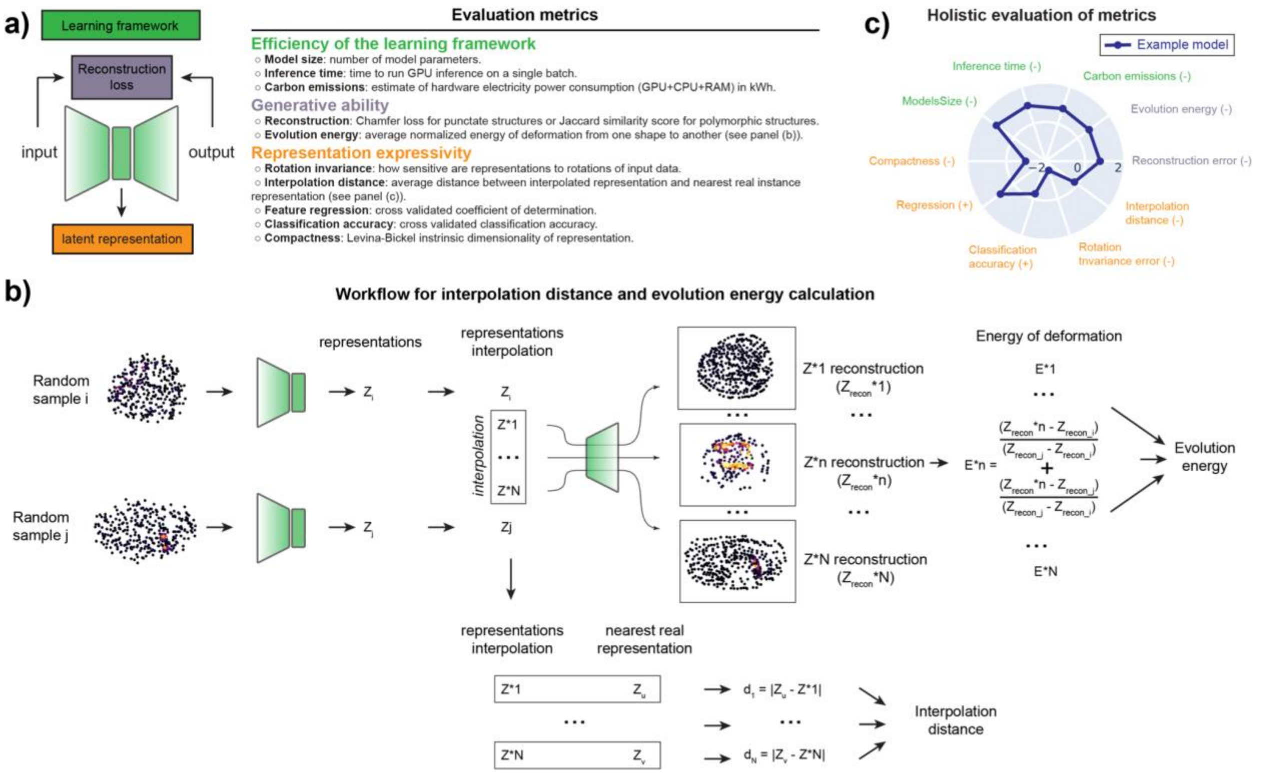

Figure S1 - Evaluation metrics for representation learning models a) Overview of different evaluation metrics for quantifying the utility of each representation learning framework. Efficiency metrics include model size, inference time, and carbon emissions. Generative ability metrics include reconstruction error and evolution energy. Representation expressivity metrics include rotation invariance error, interpolation distance, feature regression, classification accuracy, and compactness. **b)** Workflow for interpolation distance and evolution energy calculation. Two samples are drawn from the population randomly, and a linear interpolation is performed on the representations of the two samples. The euclidean distance between an interpolation and the nearest real representation is the interpolation distance. The interpolation distance is averaged across many interpolations to compute the average interpolation distance. Each interpolation is reconstructed using the decoder to obtain a reconstruction. The sum of the reconstruction error between the interpolated reconstruction and the reconstructions of the initial and final shapes normalized by the reconstruction error between the initial and final shape is the energy of deformation[7]. The energy of deformation is averaged across many interpolations to compute the evolution energy. Both evolution energy and average interpolation distance are averaged across many random pairs of samples from the test set. **c)** Holistic evaluation of metrics. Metrics are z-scored across models per metric. Z-scored metrics are visualized using a polar plot by flipping the sign for metrics where lower is better (indicated by a negative sign).

bioRxiv preprint doi: https://doi.org/10.1101/2024.07.25.605164; this version posted July 27, 2024. The copyright holder for this preprint (which was not certified by peer review) is the author/funder, who has granted bioRxiv a license to display the preprint in perpetuity. It is made available under aCC-BY 4.0 International license.

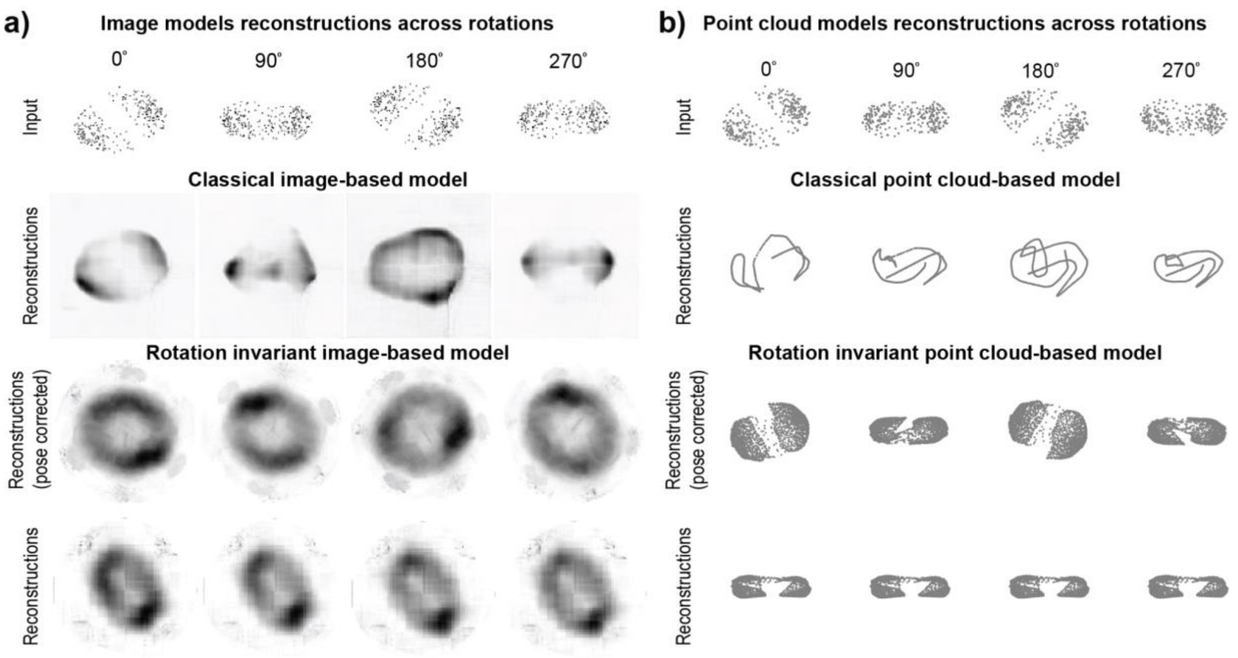

Figure S2 - Testing orientation invariance for image and point cloud models for the cellPACK synthetic dataset a) (Top row) Example image input for the planar 45 rule is rotated by four 90 degree rotations. (Middle row) Reconstructions using the classical image model (upper) and rotation invariant image model (lower) for each rotated input. The reconstructions using the rotation invariant model are pose-corrected using the learned rotation angles. (Bottom row) Rotation invariant reconstructions using the rotation invariant image model for each rotated input. **b)** (Top row) Example point cloud input for the planar 45 rule is rotated by four 90 degree rotations. (Middle row) Reconstructions using the classical point cloud model (upper) and rotation invariant point cloud model (lower) for each rotated input. The reconstructions using the rotation invariant model are pose-corrected using the learned rotation angles. (Bottom row) Rotation invariant reconstructions using the rotation invariant point cloud model for each rotated input. All reconstructions shown are max projections in Z.

bioRxiv preprint doi: https://doi.org/10.1101/2024.07.25.605164; this version posted July 27, 2024. The copyright holder for this preprint (which was not certified by peer review) is the author/funder, who has granted bioRxiv a license to display the preprint in perpetuity. It is made available under aCC-BY 4.0 International license.

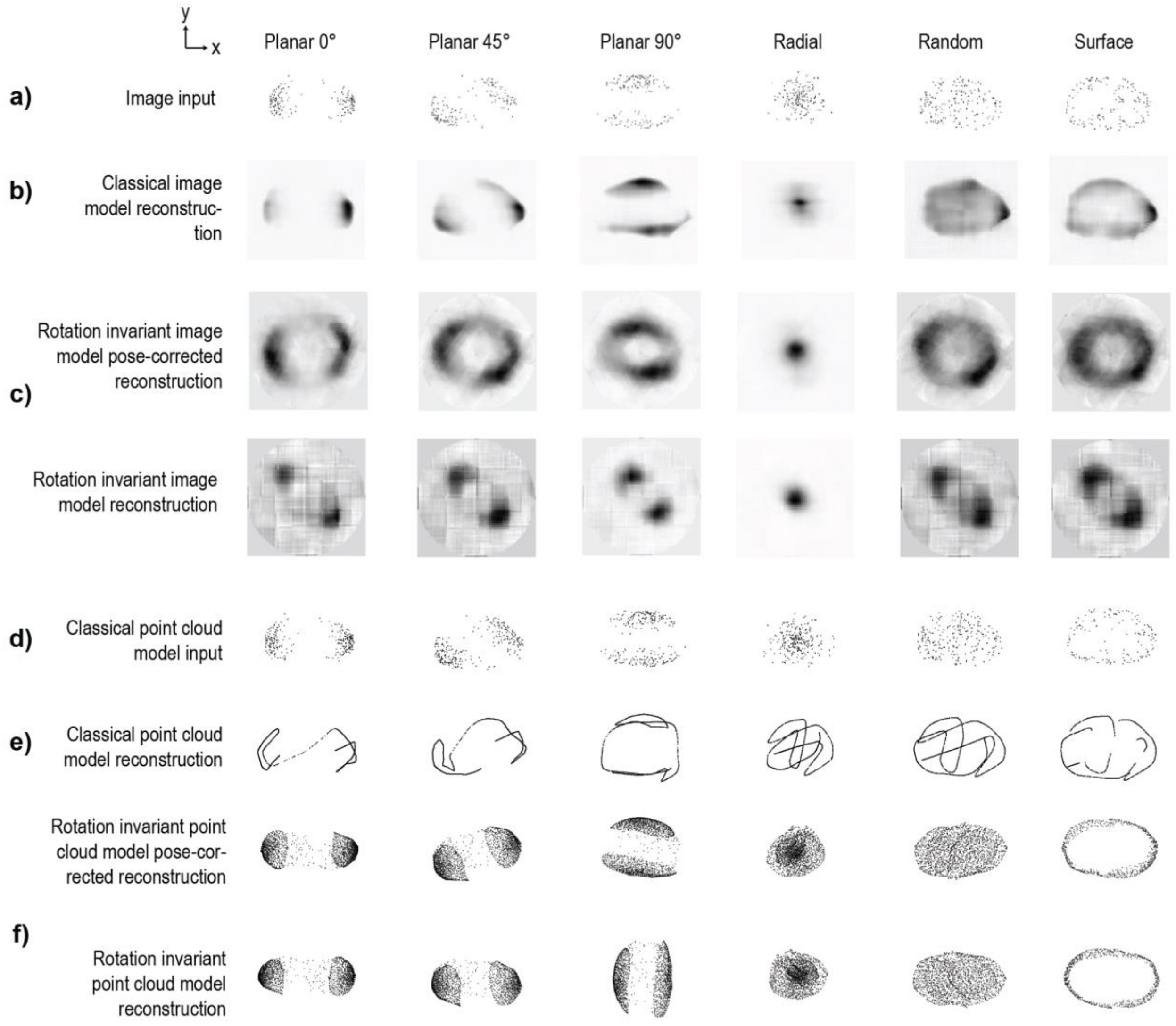

Supp Fig. 3 - **Evaluation of test set model reconstructions for synthetic punctate structures generated using cellPACK.** Test set center slice inputs (**a, d)** and reconstructions using **b)** classical image model, **c)** rotation invariant image model, **e)** classical point cloud model, and **f)** rotation invariant point cloud model for each of the 6 packing rules. Both pose-corrected and rotation invariant reconstructions are shown for the rotation invariant models.

bioRxiv preprint doi: https://doi.org/10.1101/2024.07.25.605164; this version posted July 27, 2024. The copyright holder for this preprint (which was not certified by peer review) is the author/funder, who has granted bioRxiv a license to display the preprint in perpetuity. It is made available under aCC-BY 4.0 International license.

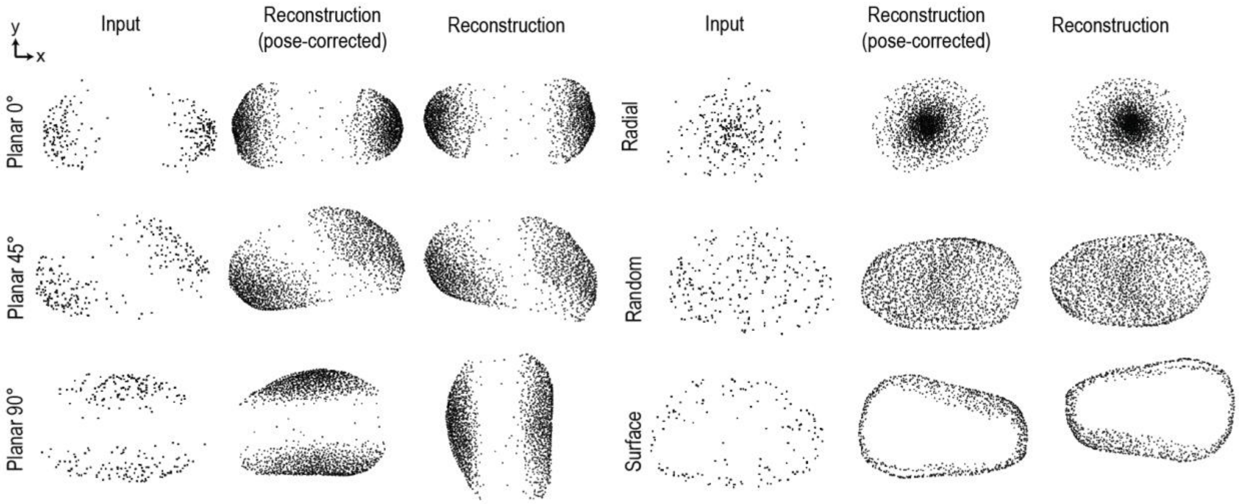

Figure S4 - Jitter augmentations slightly improve point cloud model reconstructions for synthetic punctate structures generated using cellPACK. Test set center slice reconstructions using rotation invariant point cloud model with jitter augmentations for each of the 6 packing rules.

bioRxiv preprint doi: https://doi.org/10.1101/2024.07.25.605164; this version posted July 27, 2024. The copyright holder for this preprint (which was not certified by peer review) is the author/funder, who has granted bioRxiv a license to display the preprint in perpetuity. It is made available under aCC-BY 4.0 International license.

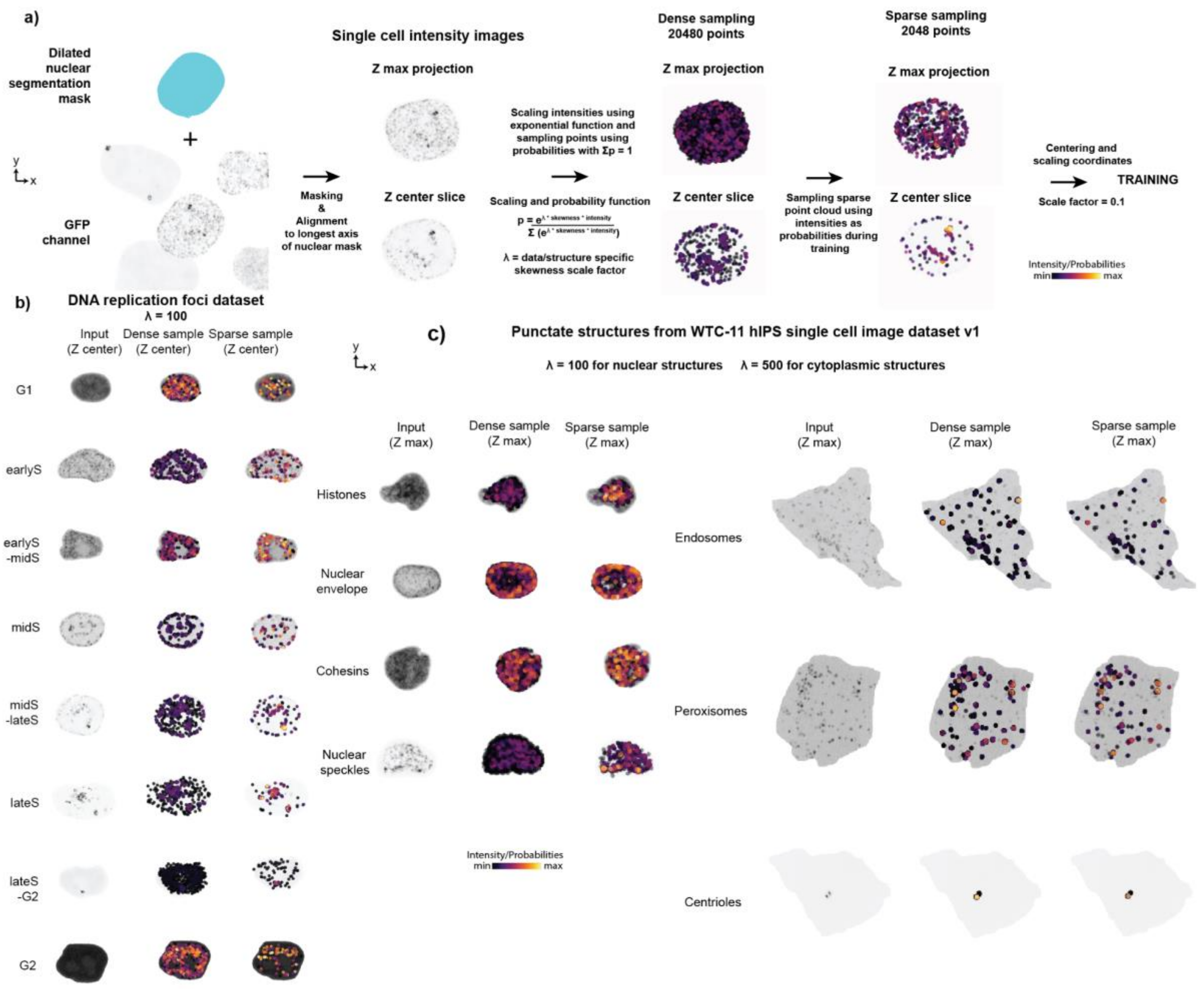

Figure S5 - 3D image preprocessing into application appropriate inputs for punctate structures. Workflow for generating 4D point clouds from 3D intensity images. **a)** single-cell intensity images are obtained by masking via a dilated nuclear mask (for nuclear structures), followed by alignment to the longest axis of the nuclear mask. Intensities were then scaled using an exponential function and then converted to probabilities. These probabilities were then used to sample a dense 4D point cloud with 20480 points and XYZ + intensity coordinates. During training, a sparse point cloud with 2048 points was sampled from this dense point cloud using the intensities as probabilities. The intensity coordinate was scaled using a scale factor of 0.1 to ensure that intensity values were in the same range as XYZ coordinate values. **b)** Examples of dense sample and sparse sample for each cell cycle stage for PCNA dataset. Shown are center-slice of raw intensity image, center-slice of raw intensity image overlaid with dense sample, and center-slice of raw intensity image overlaid with sparse training sample. **c)** Examples of dense sample and sparse sample for each punctate structure from the WTC-11 hiPSC Single-Cell Image

bioRxiv preprint doi: https://doi.org/10.1101/2024.07.25.605164; this version posted July 27, 2024. The copyright holder for this preprint (which was not certified by peer review) is the author/funder, who has granted bioRxiv a license to display the preprint in perpetuity. It is made available under aCC-BY 4.0 International license.

Dataset v1. Structures include histones, nuclear envelope, cohesins, nuclear speckles, endosomes, peroxisomes, and centrioles.

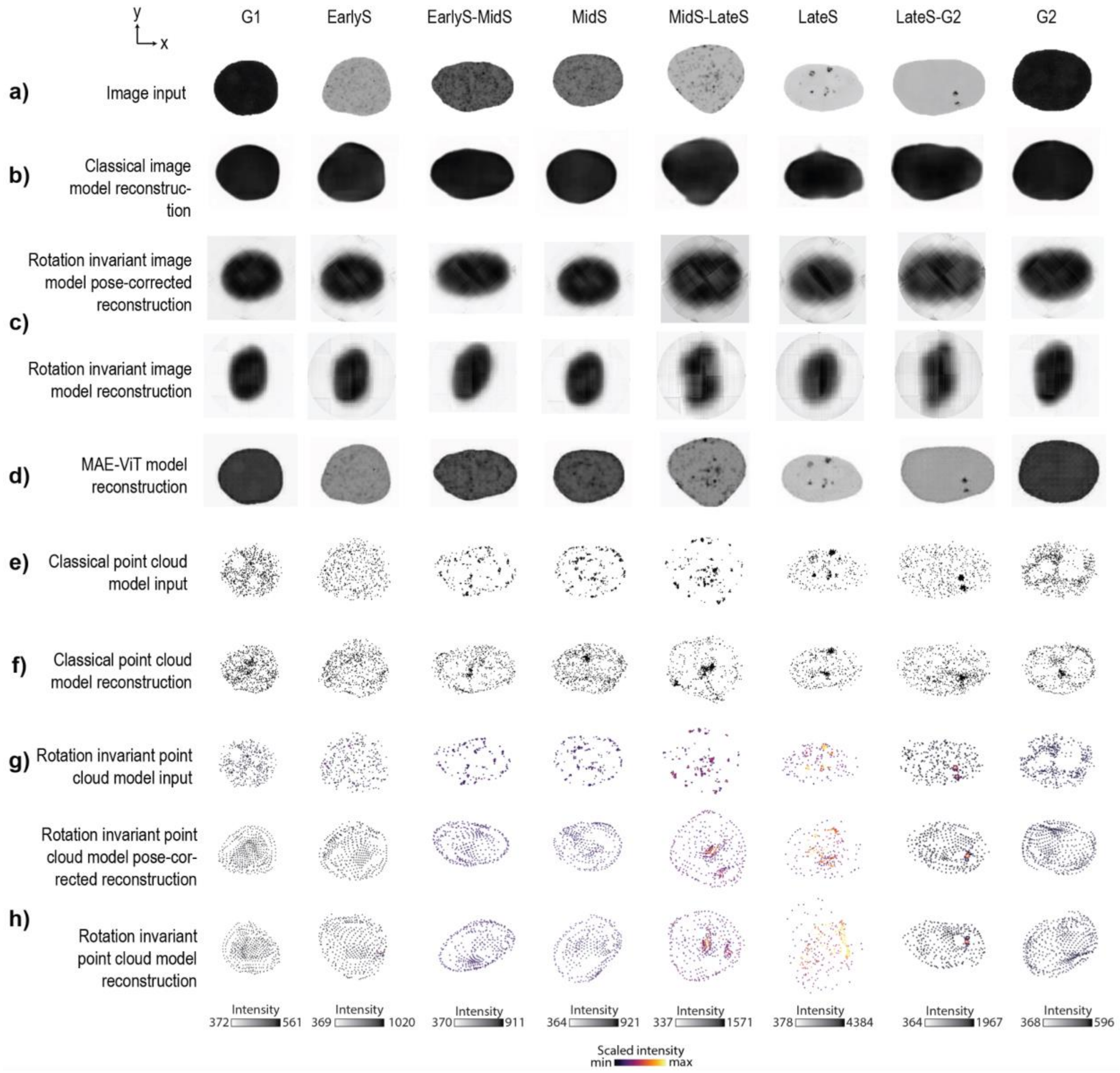

bioRxiv preprint doi: https://doi.org/10.1101/2024.07.25.605164; this version posted July 27, 2024. The copyright holder for this preprint (which was not certified by peer review) is the author/funder, who has granted bioRxiv a license to display the preprint in perpetuity. It is made available under aCC-BY 4.0 International license.

Figure S6 - Evaluation of test set model reconstructions for the DNA replication foci dataset. Test set center slice inputs (**a, e, g**) and reconstructions using **b)** classical image model, **c)** rotation invariant image model, **d)** an alternative classical image model via a masked autoencoder with a vision transformer as an encoder (MAE-ViT), **f)** classical point cloud model, and **h)** rotation invariant point cloud model for samples from each of the 8 cell cycle stages. Both pose-corrected and rotation invariant reconstructions are shown for the rotation invariant models.

bioRxiv preprint doi: https://doi.org/10.1101/2024.07.25.605164; this version posted July 27, 2024. The copyright holder for this preprint (which was not certified by peer review) is the author/funder, who has granted bioRxiv a license to display the preprint in perpetuity. It is made available under aCC-BY 4.0 International license.

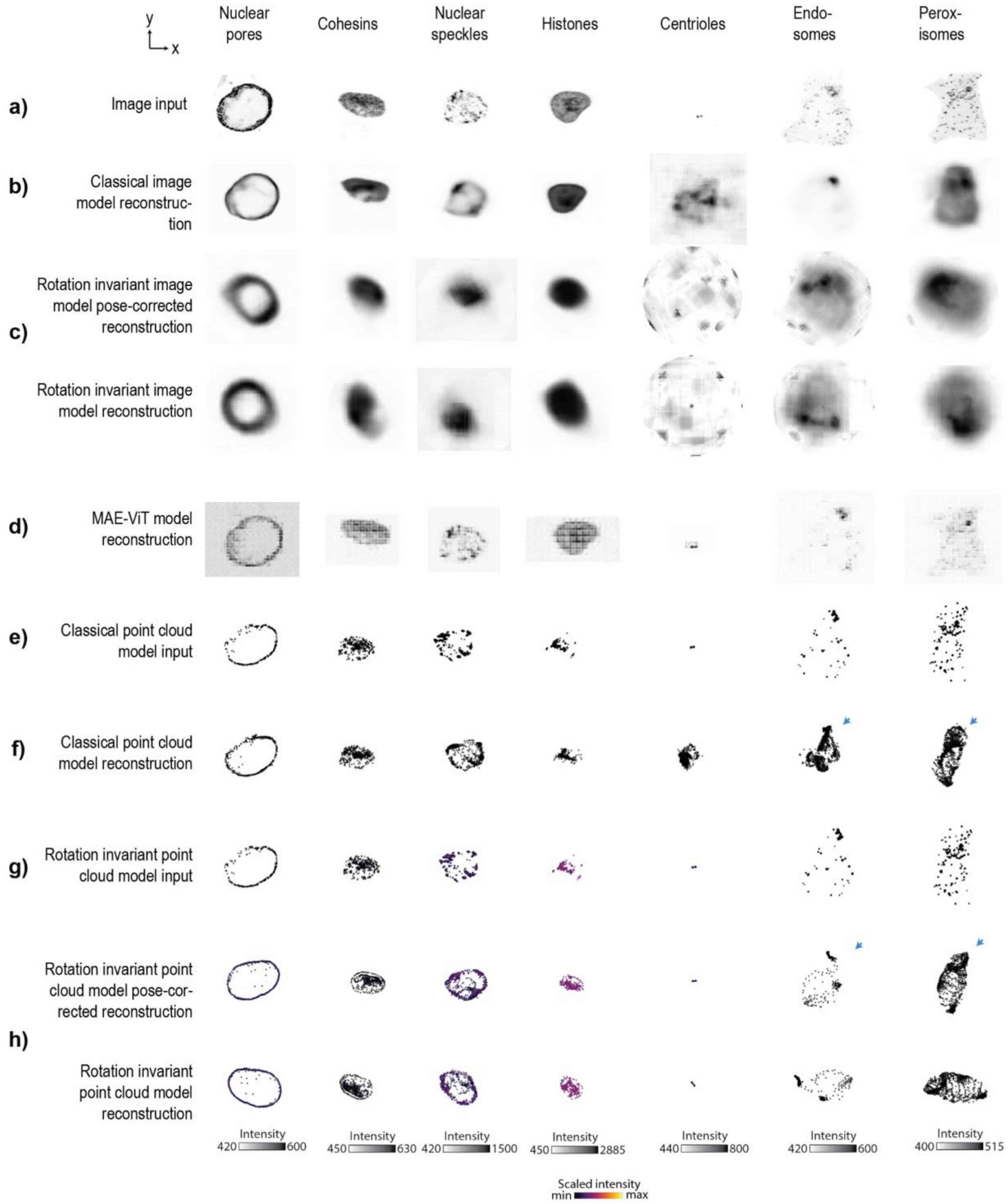

Figure S7 - Evaluation of test set model reconstructions for punctate structures from the WTC-11 hiPSC Single-Cell Image Dataset v1. Visualization of test set reconstructions for sampled histones (CellId 721646), nuclear pores (CellId 873680), cohesins (CellId 994027), nuclear speckles (CellId

bioRxiv preprint doi: https://doi.org/10.1101/2024.07.25.605164; this version posted July 27, 2024. The copyright holder for this preprint (which was not certified by peer review) is the author/funder, who has granted bioRxiv a license to display the preprint in perpetuity. It is made available under aCC-BY 4.0 International license.

490385), centrioles (CellId 451974), endosomes (CellId 811336), peroxisomes (CellId 835431). Shown are test set inputs (**a, e, g**) and reconstructions using **b)** classical image model, **c)** rotation invariant image model, **d)** an alternative classical image model via a masked autoencoder with a vision transformer as an encoder (MAE-ViT), **f)** classical point cloud model, and **h)** rotation invariant point cloud model for each structure. Both pose-corrected and rotation invariant reconstructions are shown for the rotation invariant models. Reconstructions for nuclear pores, cohesins, and histones are center slices, whereas reconstructions for nuclear speckles, centrioles, endosomes, and peroxisomes are max projections. Spatial distribution artifacts in reconstructions for endosomes and peroxisomes are highlighted with blue arrows.

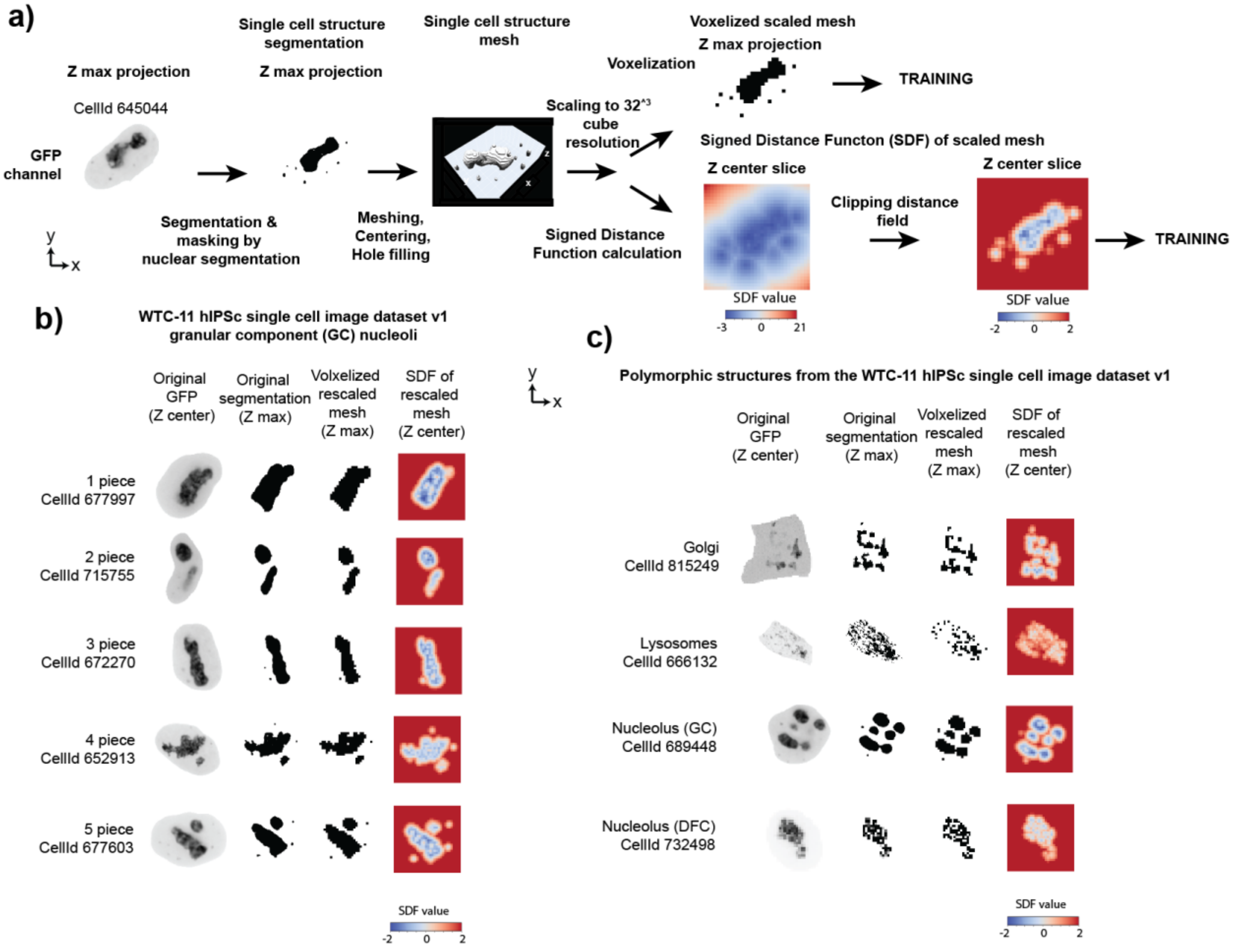

bioRxiv preprint doi: https://doi.org/10.1101/2024.07.25.605164; this version posted July 27, 2024. The copyright holder for this preprint (which was not certified by peer review) is the author/funder, who has granted bioRxiv a license to display the preprint in perpetuity. It is made available under aCC-BY 4.0 International license.

Figure S8- 3D image preprocessing into application appropriate inputs for polymorphic structures **a)** Workflow for computing signed distance function (SDF) images from segmentations. single-cell structure segmentations are masked by nuclear segmentation (for nuclear structures), followed by meshing, centering, and hole filling. The mesh is then rescaled to 32*^{3} cube resolution and then processed to get a signed distance function. Alternatively, the rescaled mesh is voxelized to get a segmentation. SDF is clipped to (-2, 2) range for training image models to focus models on the zero level set. Example shown is for nucleoli (GC). **b)**. Visualization of rescaled segmentation and SDF for examples with different numbers of pieces of granular component (GC) of nucleoli. Shown are center-slices of raw intensity images, max projection of the structure segmentation, max projection of the voxelized rescaled segmentation, and center slice of the rescaled mesh SDF. **c)** Visualization of rescaled segmentation and SDF for other polymorphic structures from the WTC-11 hiPSC Single-Cell Image Dataset v1 including lysosomes, Golgi, GC nucleoli, and dense fibrillar component (DFC) nucleoli.

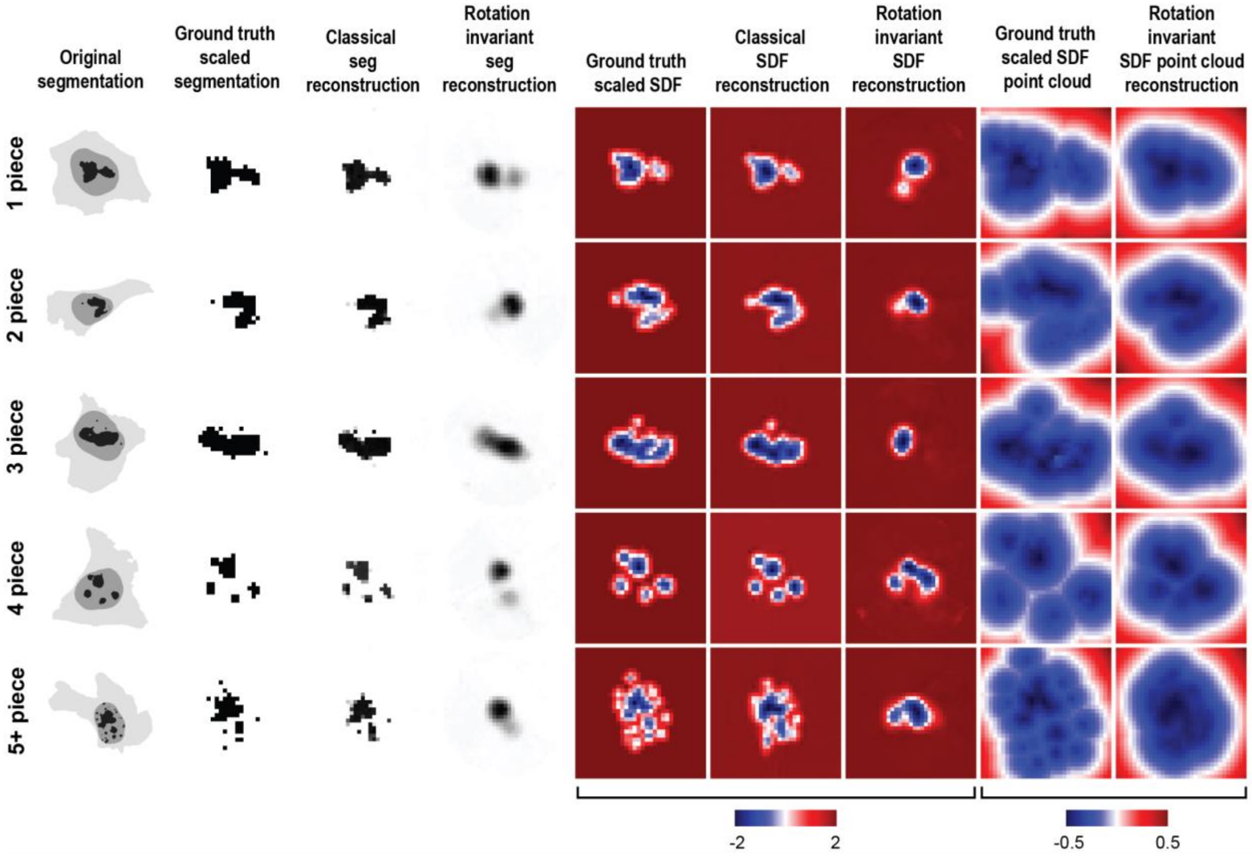

Figure S9 - Examples of model inputs and outputs for nucleolar GC dataset. Test set reconstructions across all models for sampled 1 piece (CellId 964798), 2 pieces (CellId 661110), 3 pieces (CellId

bioRxiv preprint doi: https://doi.org/10.1101/2024.07.25.605164; this version posted July 27, 2024. The copyright holder for this preprint (which was not certified by peer review) is the author/funder, who has granted bioRxiv a license to display the preprint in perpetuity. It is made available under aCC-BY 4.0 International license.

644401), 4 pieces (CellId 967887) and 5+ pieces (CellId 703621) examples. Max projections of original structure segmentations overlaid with nuclear and membrane segmentations are shown. Max projections are shown for segmentations, whereas middle slices are shown for SDFs.

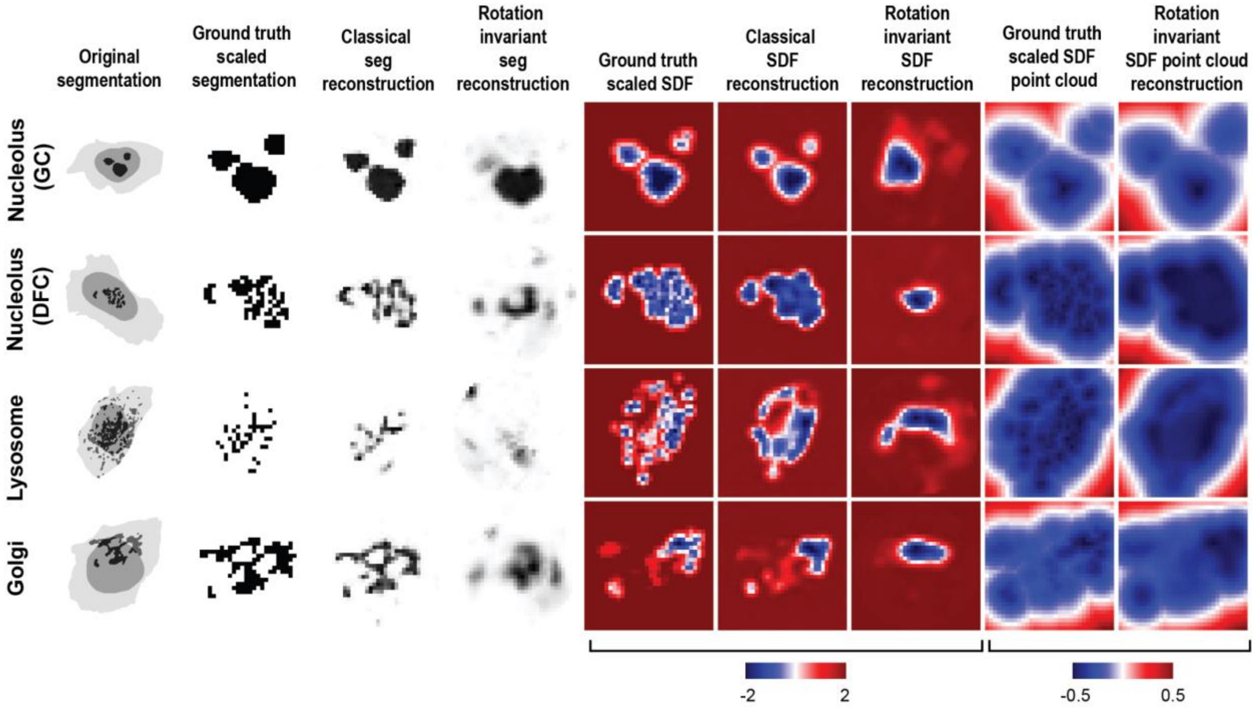

Figure S10 - Evaluation of model reconstructions for polymorphic structures from the WTC-11 hiPSC Single-Cell Image Dataset v1. A) Test set reconstructions across all models for sampled nucleoli (GC) (CellId 691110), nucleoli (DFC) (CellId 723687), lysosome (CellId 816468), and Golgi (CellId 800894) examples. Max projections of original structure segmentations overlaid with nuclear and membrane segmentations are shown. Max projections are shown for segmentations, whereas middle slices are shown for SDFs.
